# Identification and Characterization of Highly Divergent Simian Foamy Viruses in a Wide Range of New World Primates from Brazil

**DOI:** 10.1371/journal.pone.0067568

**Published:** 2013-07-03

**Authors:** Cláudia P. Muniz, Lian L. Troncoso, Miguel A. Moreira, Esmeralda A. Soares, Alcides Pissinatti, Cibele R. Bonvicino, Héctor N. Seuánez, Bechan Sharma, Hongwei Jia, Anupama Shankar, William M. Switzer, André F. Santos, Marcelo A. Soares

**Affiliations:** 1 Departamento de Genética, Universidade Federal do Rio de Janeiro, Rio de Janeiro, Rio de Janeiro, Brazil; 2 Programa de Genética, Instituto Nacional de Câncer, Rio de Janeiro, Rio de Janeiro, Brazil; 3 Centro de Primatologia do Rio de Janeiro, Rio de Janeiro, Rio de Janeiro, Brazil; 4 Instituto Oswaldo Cruz, Rio de Janeiro, Brazil; 5 Department of Biochemistry, University of Allahabad, Allahabad, India; 6 Division of HIV/AIDS Prevention, Center for Disease Control and Prevention, Atlanta, Georgia, United States of America; University of Florida, United States of America

## Abstract

Foamy viruses naturally infect a wide range of mammals, including Old World (OWP) and New World primates (NWP), which are collectively called simian foamy viruses (SFV). While NWP species in Central and South America are highly diverse, only SFV from captive marmoset, spider monkey, and squirrel monkey have been genetically characterized and the molecular epidemiology of SFV infection in NWPs remains unknown. We tested a large collection of genomic DNA (n  = 332) comprising 14 genera of NWP species for the presence of SFV polymerase (*pol*) sequences using generic PCR primers. Further molecular characterization of positive samples was carried out by LTR-*gag* and larger *pol* sequence analysis. We identified novel SFVs infecting nine NWP genera. Prevalence rates varied between 14–30% in different species for which at least 10 specimens were tested. High SFV genetic diversity among NWP up to 50% in LTR-*gag* and 40% in *pol* was revealed by intragenus and intrafamilial comparisons. Two different SFV strains infecting two captive yellow-breasted capuchins did not group in species-specific lineages but rather clustered with SFVs from marmoset and spider monkeys, indicating independent cross-species transmission events. We describe the first SFV epidemiology study of NWP, and the first evidence of SFV infection in wild NWPs. We also document a wide distribution of distinct SFVs in 14 NWP genera, including two novel co-speciating SFVs in capuchins and howler monkeys, suggestive of an ancient evolutionary history in NWPs for at least 28 million years. A high SFV genetic diversity was seen among NWP, yet these viruses seem able to jump between NWP species and even genera. Our results raise concerns for the risk of zoonotic transmission of NWP SFV to humans as these primates are regularly hunted for food or kept as pets in forest regions of South America.

## Introduction

Foamy viruses (FV) are complex retroviruses in the Spumavirus genus that naturally infect a wide range of mammals, including bovines, felines, equines, sheep, and nonhuman primates (NHPs). In NHPs, FV are referred to as simian foamy viruses (SFV). Their unusual name refers to the formation of syncytia of multinucleated giant cells with numerous vacuoles seen by electron microscopy with a foamy appearance upon virus infection *in vitro* [1]. Although highly cytopathic in tissue culture, FV is apparently nonpathogenic *in vivo*, but a clear causality between infection and disease has not been systematically investigated either in animals or in humans with zoonotic SFV infection [2].

While SFV isolates have been identified in numerous, diverse species of African and Asian NHPs [3]–[7], or Catarrhini, humans do not appear to be a natural host of these viruses. All SFVs detected in humans were acquired by zoonotic transmission from infected African and Asian NHPs and thus far are persistent but seemingly asymptomatic infections [2], [8]–[12]. The zoonotic transmission of SFV to humans in the wild is not a rare event and the presence of FV ranges from approximately 1% in persons having contact with NHPs through hunting, butchering and keeping NHP pets, to as high as 19% in hunters with severe mucocutaneous exposures [13]–[17]. Persons infected with SFV while working with NHPs in biomedical facilities have also been reported with prevalence ranging from 3–4% [9], [14], [15].

Despite the wide distribution and diversity of SFV in different Old World primate species, studies of SFV in New World primates (NWP), or Platyrrhini, have been limited to very small numbers of captive animals. NWP are a diverse group of American primates, comprising over 110 different species in 15–19 genera and three families (Atelidae, Aotidae, Cebidae, and Pitheciidae) [18], [19]. However, molecular characterization of SFV has been reported for only three species of captive NWP, *Ateles sp*. (spider monkey), *Saimiri sciureus* (common squirrel monkey) and *Callithrix jacchus* (common marmoset), representative of only two families (Atelidae and Cebidae), with complete genomes only available recently [20], [21]. Thus, the prevalence of SFV in NWPs, especially in wild animals remains largely unknown. NWPs are commonly kept as pets in several countries because of their small size and are also hunted and butchered for consumption in South America, resulting in potential human exposure to SFV. Similarly, NWPs are commonly used in biomedical research studies placing animal handlers, veterinarians, and scientists at increased risk of exposure to SFV. Determining the prevalence and geographical distribution of SFV in NWP is the first step to better understand the public health risk of infection with SFV in persons exposed to NWPs.

Here we identify SFV infection in a wide range of NWP species from all three NWP families, including the molecular characterization of highly divergent SFV in capuchin (*Cebus* species) and howler (*Alouatta* sp.) monkeys. We also report for the first time SFV infection in wild NWPs and evidence for independent cross-species transmission of SFV in two different captive capuchins (*Cebus xanthosternos*) likely originating from spider monkey (*Ateles* sp.) and marmoset (*Callithrix jacchus*). Our results highlight the need to further characterize the geographic distribution and evolutionary history of SFV in NWPs from other South American countries. In addition, our results emphasize the need to define the risk of infection with SFV in persons exposed to NWPs.

## Materials and Methods

### Ethics Statement

**Figure 1:**
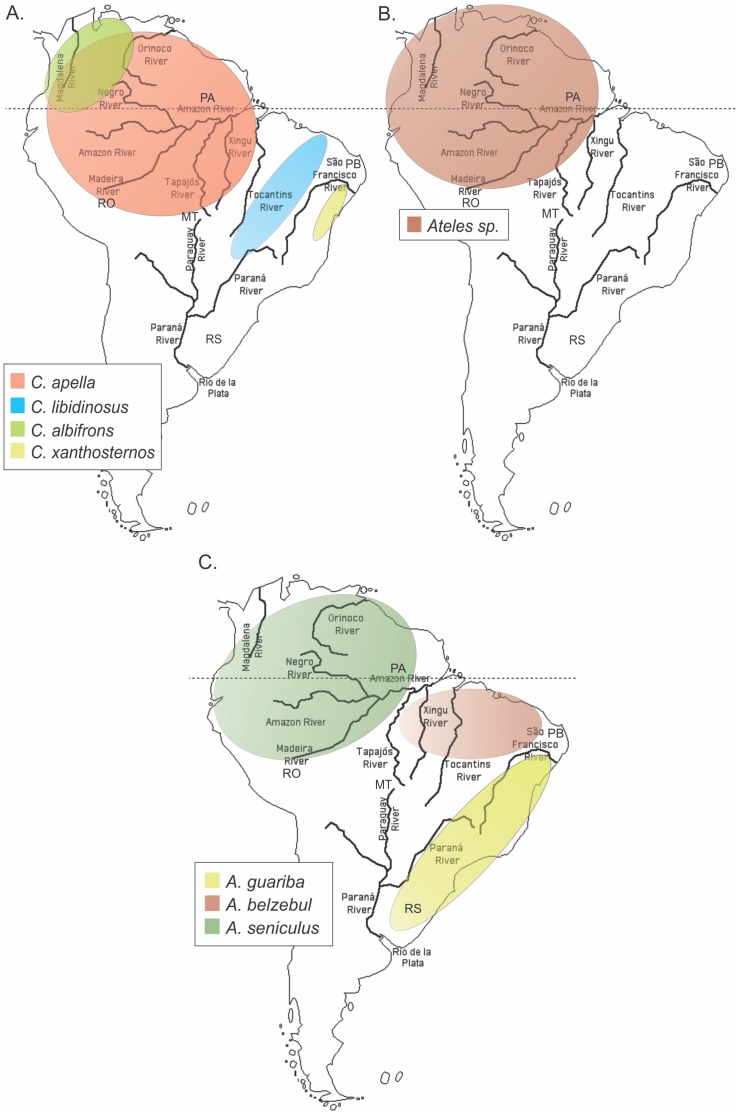
Geographic distribution of distinct *Cebus* (*A*), *Ateles* (*B*) and *Alouatta* (*C*) primate species in Brazil. Data are according to the Database of Georeferenced Occurrence Localities of Neotropical Primates, Department of Zoology, Universidade Federal de Minas Gerais, Brazil (http://www.icb.ufmg.br/zoo/primatas/home_bdgeoprim.htm). Primate center and wild animal site locations within Brazil (see Table 4) are shown: Pará (PA), Mato Grosso (MT), Rondônia (RO), Paraíba (PB), and Brazil/Argentina frontier (RS).

Original NWP samples have been previously collected by venous puncture (n  = 462) or by liver biopsy of dead animals (n  = 9) following the national guidelines and provisions of IBAMA (Instituto Brasileiro do Meio Ambiente e dos Recursos Naturais Renováveis, Brazil; permanent license number 11375–1), which included animal welfare standard operational procedures. All samples (wild and captivity) have been collected by some of the authors (EAS, MAM, AP, CRB and HNS) for previous host phylogenetic and cytogenetic studies [22]–[25]. All nine liver samples were from animals sacrificed during a survey of *Trypanosoma* reservoirs in the northern states of Pará and Rondônia (see Table 1 and Figure 1 for specific geographic locales). Sacrifice of these animals was conducted by anesthetic lethal dose injection following the guidelines of the Brazilian Council of Biology (http://www.cfbio.gov.br). Specimens from wild animals were collected during their rescue and relocation in forest areas to be flooded for construction of hydroelectric power houses in northern Brazil (see Table 1 and Figure 1 for specific collection locales). Animals were caught and subject to ketamine anesthesia for venous puncture, followed by relocation to another area of the same habitat.

**Table 1:** SFV PCR prevalence in NWP living at primate centers and in the wild^a^.

Species	Origin^b^	State/location in Brazil/South America^c^	# pos/total	(%)
***Alouatta belzebul***	UHE Tucuruí (wild)	Pará (PA)	13/45	29%
***Alouatta caraya***	UHE Manso (wild)	Mato Grosso (MT)	6/20	30%
***Aotus azarai***	CNP	Rondônia (RO)	2/9	22%
		Pará (PA)	2/9	22%
***Cebus apella***	CPB	Paraíba (PB)	3/22	14%
	CEMIC	Frontier Brazil/Argentina (RS)	3/19	16%

a PCR testing using diagnostic primers to detect 192-bp polymerase sequences in DNA specimens from species listed;

b UHE, Usina Hidroelétrica; CNP, Centro Nacional de Primatologia; CPB, Centro de Proteção de Primatas Brasileiros, Instituto Chico Mendes de Conservação da Biodiversidade; CEMIC, Centro de Educación Médica e Investigaciones Clínicas Norberto Quirno;

c N, north; SE, southeast; NE, northeast; S, south.

### NWP Specimen Preparation and Species Confirmation

Genomic DNA (gDNA) samples were extracted from 471 NWPs comprising 14 different genera and were stored at –20°C at the Primate Genomic DNA Bank of the Instituto Nacional de Câncer, located in Rio de Janeiro, Brazil. These specimens were previously collected from NWPs in the wild (n  = 65) and in captivity (n  = 406) from the Brazilian National Primate Center, the Rio de Janeiro Primate Center, the Brazilian Primate Protection Center, the Center for Medical Education and Clinical Investigation Norberto Quirno and the Zoos of Rio de Janeiro and São Paulo, as described elsewhere [22]–[25] and comprised whole blood (n  = 462) or liver (n  = 9) biopsies. Locations of the five study sites in Brazil are shown in Fig. 1. DNA was extracted using the Qiagen DNA genomic DNA extraction kit (Qiagen, Chatsworth, CA), according to the manufacturer’s specifications, or by standard phenol-chloroform techniques.

Genomic DNA integrity (and suitability for SFV proviral detection) and primate host species taxonomic classification were determined by analysis of a 975–bp cytochrome B (*cytB*) mitochondrial sequence obtained by one-step PCR using primers L14724 (5′CGA AGC TTG ATA TGA AAA ACC ATC GTT G 3′) and Mus15398 (5′GAA TAT CAG CTT TGG GTG TTG RTG 3′) [26]. Through this analysis, 332 samples (70.5%) were further considered suitable for SFV detection (Table 2). *cytB* sequences from SFV-positive specimens (see below) were aligned with those available from NWP at GenBank and trees were inferred with maximum likelihood (ML) using MEGA v5.0 [27]. The HKY+I+G model of nucleotide substitution was inferred using the ML method with goodness of fit measured by the Bayesian information criterion in MEGA v.5.0. Cytochrome B sequences from a chimpanzee (*Pan troglodytes*, GenBank accession number EF660790) and an African green monkey (*Chlorocebus aethiops*, GenBank accession number AB495295) were used as outgroups for the phylogenetic analyses.

**Table 2:** Molecular detection and distribution of SFV in New World primates from Brazil.

Family	Scientific name	Common name^a^	No. pos/No. total (%)^b^
**Atelidae**	*Alouatta belzebul*	black-and-red howler monkey	13/45 (29) c
	*Alouatta belzebul*		2/5 (40)
	*Alouatta caraya*	black howler monkey	6/20 (30)
	*Alouatta guariba*	brown howler monkey	2/3 (66.7)
	*Alouatta seniculus*	red howler monkey	4/8 (50)
	*Ateles paniscus*	black spider monkey	0/1 (0)
	*Brachyteles arachnoides*	wooly spider monkey	0/1 (0)
	*Lagothrix species*	wooly monkey	0/2 (0)
**Cebidae**	*Aotus azarai*	Azara’s owl monkey	4/28 (14.3)
	*Aotus species*	owl monkey	2/24 (8.3)
	*Callimico goeldii*	Goeldi’s marmoset	0/2 (0)
	*Callithrix argentata*	silvery marmoset	3/8 (37.5)
	*Callithrix aurita*	white-eared marmoset	1/1 (100)
	*Callithrix emiliae*	Emilia’s marmoset	3/9 (33.3)
	*Callithrix geoffroyi*	Geoffroy’s marmoset	0/1 (0)
	*Callithrix humeralifera*	Santarem marmoset	0/3 (0)
	*Callithrix jacchus*	white-tufted-ear marmoset	1/1 (100)
	*Callithrix kuhlii*	Wied’s marmoset	0/3 (0)
	*Callithrix melanura*	black-tailed marmoset	1/2 (50)
	*Callithrix penicillata*	black-pencilled marmoset	0/1 (0)
	*Cebus albifrons*	white-fronted capuchin	3/13 (23.1)
	*Cebus apella*	tufted capuchin	11/50 (22)
	*Cebus cay*	hooded capuchin	0/7 (0)
	*Cebus olivaceus*	weeper capuchin monkey	6/16 (37.5)
	*Cebus xanthosternos*	yellow-breasted capuchin	5/9 (55.6)
	*Cebus species*	Capuchin	0/3 (0)
	*Leontopithecus chrysomelas*	golden-and-black lion tamarin	0/1 (0)
	*Leontopithecus chrysopygus*	golden-rumped lion tamarin	0/1 (0)
	*Leontopithecus rosalia*	golden lion tamarin	1/2 (50)
	*Saguinus fuscicollis*	brown-headed tamarin	0/2 (0)
	*Saguinus imperator*	Emperor tamarin	2/2 (100)
	*Saguinus martinsi*	Martin’s bare-face tamarin	0/1 (0)
	*Saguinus midas*	Midas tamarin	0/3 (0)
	*Saguinus mystax*	moustached tamarin	0/1 (0)
	*Saguinus niger*	black-handed tamarin	0/1 (0)
	*Saimiri sciureus*	common squirrel monkey	3/17 (17.6)
	*Saimiri ustus*	bare-eared squirrel monkey	4/16 (25)
	*Saimiri species*	squirrel monkey	1/3 (33.3)
**Pitheciidae**	*Callicebus moloch*	red-bellied titi	1/1 (100)
	*Callicebus nigrifrons*	black-fronted titi	0/3 (0)
	*Callicebus personatus*	masked titi	0/3 (0)
	*Callicebus torquatus*	yellow-handed titi	0/2 (0)
	*Callicebus species*	titi monkey	0/1 (0)
	*Chiropotes species*	bearded saki monkey	1/3 (33.3)
	*Pithecia irrorata*	bald-faced saki	0/3 (3)
**Total**			80/332 (24.1)

a NWP common names are as in [19].

b PCR testing using diagnostic primers to detect 192-bp polymerase sequences in DNA specimens from species listed.

c Underlined numbers refer to specimens from the wild.

### SFV PCR and Sequence Analysis

All DNA samples were first screened for 192-bp SFV sequences using a novel semi-nested PCR that utilizes generic polymerase gene (*pol*) primers (Table S1) and conditions previously reported for other generic SFV *pol* PCR [9]. These primers were designed using an alignment of sequences from the three complete NWP SFV genomes available at GenBank from marmoset, squirrel, and spider monkey (accession numbers GU356395, GU356394, and EU010385, respectively) [20], [21].

To analyze the phylogenetic relationships with other previously described NWP SFV, two additional SFV subgenomic regions were PCR-amplified and sequenced. Generic primers for this additional testing were designed using conserved regions in an alignment of the three complete NWP SFV genomes [20], [21] to amplify 398-bp LTR and *gag*-matrix (225-bp in LTR and 173-bp in *gag*) and 520-bp *pol* sequences using nested PCR (Table S1). Amplified products were purified, quantified, and sequenced on both strands using the Big Dye v.3.1 kit (Life Technologies, Carlsbad, USA) and an automated ABI 3130XL Genetic Analyzer and edited with SeqMan v7.0 (DNASTAR, Madison, USA). New sequences were aligned with those available from NWP retrieved from GenBank. Nucleotide mean genetic distances were calculated within NWP and OWP SFV, within NWP families (Atelidae and Cebidae) and within different NWP genera using the pairwise distance tool in MEGA5 with Kimura’s 2-parameter model of nucleotide substitution.

Phylogenetic inference of the LTR-*gag* and *pol* sequences was conducted using neighbor-joining (NJ), and ML methods implemented in MEGA v.5.0 using either the Tamura-Nei or HYY+I+G nucleotide substitution models inferred in MEGA v5.0. SFV sequences from a chimpanzee (SFVcpz; GenBank accession number U04327) and an African green monkey (GenBank accession number NC_010820) were used as outgroups for the phylogenetic analyses. Phylogenetic signal in the alignments was assessed using likelihood mapping in TreePuzzle v.5.2 [28]. Nucleotide substitution saturation was measured using the method of Xia *et. al.* [29] and by transition (Ts) and transversion (Tv) *versus* divergence plots in the program DAMBE V5.0 [29].

### Phylogenetic and Molecular Dating Estimates of SFV and Host Divergences

Phylogenies and divergence time estimates for the SFV pol and host cytB sequences were inferred simultaneously using Bayesian methods in BEAST v1.6.2. For SFV, a 276-bp alignment of the larger *pol* sequences from 12 Brazilian NWMs and those from SFVmar, SFVsqu, SFVspm, SFVcpz, and SFVagm were used. Similarly, a 500-bp alignment of 15 Brazilian NWM and 16 reference *cytB* sequences was used to infer the host divergence dates and phylogeny. An uncorrelated lognormal relaxed-clock, HKY+I+G DNA substitution model with four rate categories, and Yule process of speciation tree prior were used for the analyses. Two normal priors based on primate fossil evidence were used as calibration points to infer the SFV and host divergence dates; a mean of 43.0 million years ago (MYA) with a standard deviation of 4.5 for the time to most recent common ancestor (tMRCA) of Haplorrhini and a mean of 29.0 MYA and standard deviation of 6.0 for the Catarrhini tMRCA as described elsewhere [30]. Two independent MCMC runs of 80–160 million chains each were performed to ensure convergence of the sampling which was checked in the program Tracer for effective sample sizes (ESS) >150. Trees were saved every 8,000–16,000 generations and the tree with the maximum product of the posterior clade probabilities (maximum clade credibility tree) was chosen from the posterior distribution of 10,001 sampled trees after burning in the first 1,000 sampled trees with the program TreeAnnotator v.1.6.2. Node heights were calculated from the posterior distribution of the trees. Trees were viewed in FigTree v.1.3.1. Testing of the molecular clock evolution of the SFV *pol* and *cytB* sequences was carried out using the ML method in MEGA5 with the inferred ML trees.

### SFV and Simian Host Co-evolution Inference

Reconciliation analysis and comparison of branch lengths and coalescence times of the SFV and *cytB* Bayesian trees were performed with the TreeMap (v1.0) program in accordance with the author’s instructions [31]. The significance of the observed fit between the SFV and primate trees and branch lengths was determined by comparison with the distribution of the same measure of fit for 10,000 randomly generated trees or branch lengths by using the proportional-to-distinguishable model of the randomization test incorporated in TreeMap.

#### GenBank accession numbers

All SFV and *cytB* sequences generated herein have been deposited at GenBank with the accession numbers KC331071 to KC331109.

## Results

### High Prevalence and Broad Distribution of SFV in NWP

A novel PCR assay was developed to generically detect SFV *pol* sequences in a variety of NWPs using an alignment of complete SFV genomes from marmoset, squirrel, and spider monkeys. The assay was validated using peripheral blood lymphocyte DNA from 47 seronegative and 59 seropositive NWPs identified using a Western blot (WB) test that utilizes SFV antigens from marmoset and spider monkeys grown in Cf2Th cells. Details of the SFV WB test are similar to those previously published with the exception that the previous assay utilizes two antigens each from an SFV-infected OWM or ape [32]. The 105 NWPs used for the PCR assay validation were all housed at various US institutions. gDNA was available from seven genera of NWPs including *Cebus, Alouatta, Callithrix, Aotus, Ateles, Saimiri, Cacajao* and *Pithecia*. The PCR assay had a sensitivity of 100% (92–100%; 95%CI) and detected SFV sequences in all 59 WB-positive animals. Forty-three out of the forty-seven WB-negative animals were PCR negative, giving an assay specificity of 91% (79–97%; 95% CI). The lower assay specificity results from DNA samples from four *Saimiri* specimens that were repeatedly PCR-positive but WB-negative using samples collected 2 years apart, suggestive of latent infection. These data have been reported previously [33].

**Figure 2:**
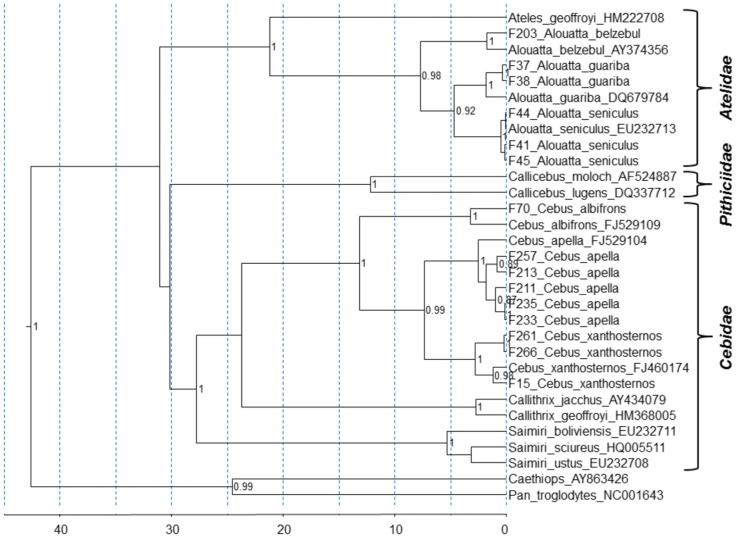
Taxonomical classification of SFV-positive NWP specimens based on phylogenetic inference of 500-bp cytochrome B (*cytB*) sequences. GenBank accession numbers of reference *ctyB* sequences are provided. Topology and divergence dates were inferred using a relaxed molecular clock and a Yule tree prior using BEAST v1.6.2. X axis is in millions of years. Posterior probabilities >0.8 are provided at nodes.

The shorter SFV *pol* sequences were detected in 80 of the 332 (24.1%) NWP DNA specimens using the generic PCR screening assay. As shown in Table 2, nine distinct genera and at least 19 different species, including representatives of all three NWP families according to the *cytB* phylogenetic relationships of the Platyrrhini group [30], had detectable SFV integrated into their genomes. Phylogenetic comparison of new *cytB* sequences from 15 Brazilian primates with those from 16 reference sequences identified five *Cebus apella*, three *Cebus xanthosternos*, one *Cebus alibrons*, three *Alouatta seniculus*, two *Alouatta guariba*, and one *Alouatta belzebul* (Fig. 2). Species classification for 36 specimens from NWPs of the *Aotus* (n  = 24), *Lagothrix* (n  = 2), *Cebus* (n  = 3), *Saimiri* (n  = 3), *Callicebus* (n  = 1), and *Chiropotes* (n  = 3) genera could not be molecularly confirmed due to the lack of representative *cytB* sequences from these species in GenBank for comparison. However, classification of NWPs in our study also included morphological characteristics typical of the species recorded by experienced taxonomists from our group. SFV prevalence ranged from 0–100% in the total study population but the extreme range may likely reflect the low numbers of samples from some species (Table 2). However, when species with less than 10 representatives are excluded from the analysis, the SFV prevalence is 23.1% (54/234) which remains similar to the rate for the total population. Two specimens were found to be SFV-positive from liver biopsies, suggesting that this virus can also be retrieved from that body compartment. Similar proportions of SFV-positive samples were found from whole blood and liver biopsies (2 and 2.5%, respectively), providing evidence for no particular bias in SFV detection when comparing both compartments.

All specimens surveyed were adults, and similar numbers of males and females were found among the SFV-positive and SFV-negative animals (56∶44% and 51∶49%, respectively), as it has been seen in other studies. Ages at specimen collection were not available for the majority of animals limiting an assessment of age-related SFV restriction in our study.

To obtain a preliminary estimate of SFV prevalence in wild and captive NWP, we determined the presence of SFV in four NWM species groups for which we had specimens from at least 10 different animals housed at primate centers or living in the wild. Overall, the molecular prevalence ranged between 14 and 29% (Table 1). Prevalence rates were consistently similar in populations from the wild or in captivity.

### Diversity and Co-evolution of SFV in NWPs

Although an alignment of the shorter *pol* sequences (192-bp PCR product, 138-bp final alignment) was shown to contain adequate phylogenetic signal by likelihood mapping and lacked substitution saturation by the method of Xia *et al*. [29], only 44/138 (32%) sites were fully resolved and all three codon positions were found to have substitution saturation in Ts and Tv *versus* divergence plots. Indeed, the inferred phylogenies were poorly resolved with weak support at most nodes which contained mixtures of SFV from multiple species atypical of SFV evolution (Fig. S1). One clear example is the clustering of SFV *pol* sequences from an African green monkey (agm) and chimpanzee (cpz) within a NWM clade which have been shown previously to form distinct phylogenetic clades [18] (Fig. S1). Thus, we amplified longer *pol* (520-bp) and LTR-*gag* sequences to better resolve the SFV phylogenies and compared these to that of the host *cytB* sequences to infer possible co-evolutionary histories.

Genetic diversity in NWP SFVs was investigated by comparing the divergence present in the new SFV sequences generated in our study to each other and to other SFV from OWP and NWP (Table 3). The nucleotide diversity of SFVcap (within capuchins) was 6–9% in both regions analyzed. The nucleotide diversity within SFV infecting members of the Cebidae (SFVcap and SFVmar) and of the Atelidae (SFVhow and SFVspm) families was similar in the *gag* and *pol* fragments (41%) and slightly higher in the LTR region (50%). We observed that the genetic diversity in *pol* was almost three times that in SFV infecting the Atelidae family than SFV infecting Cebidae. The nucleotide diversity between NWP and OWP SFV was very high, 80% in *gag*, the most divergent major gene among SFV, and 54% in *pol* (Table 3).

**Table 3:** Intra- and inter-primate family and order SFV nucleotide diversitya,b,c.

	Intra Cebidae Family		Intra Atelidae Family		Intra NWP Order	Intra OWP Order^d^	NWP X OWP
	within SFVcap	SFVcap X SFVmar	within SFVhow	SFVhow X SFVspm	Cebidae X Atelidae		
**LTR (225-bp)**	0.077 (0.047)	0.217 (0.012)	0.150 (0.056)	0.254 (0.013)	0.503 (0.035)	0.180 (0.044)	0.581 (0.084)
***gag*** ** (157-bp)**	0.091 (0.036)	0.188 (0.009)	0.192 (0.024)	0.265 (0.017)	0.409 (0.029)	0.698 (0.177)	0.802 (0.108)
***pol*** ** (347-bp)**	0.063 (0.019)	0.124 (0.007)	0.172 (0.024)	0.311 (0.010)	0.410 (0.028)	0.383 (0.067)	0.544 (0.034)

a Nucleotide diversity calculated using pairwise distances implemented in MEGA5, numbers in parentheses are standard deviations from the mean diversity;

b cap, capuchin; mar, marmoset (GenBank accession number GU356395); spm, spider monkey (EU010385);

c NWP, New World primate; OWP, Old World primate sequences included.

d For this analysis we used SFVgor [GenBank accession # HM245790], SFVora [AJ544579], SFVcpz [U04327], SFVmac [X54482] and SFVagm [M74895].

**Figure 3:**
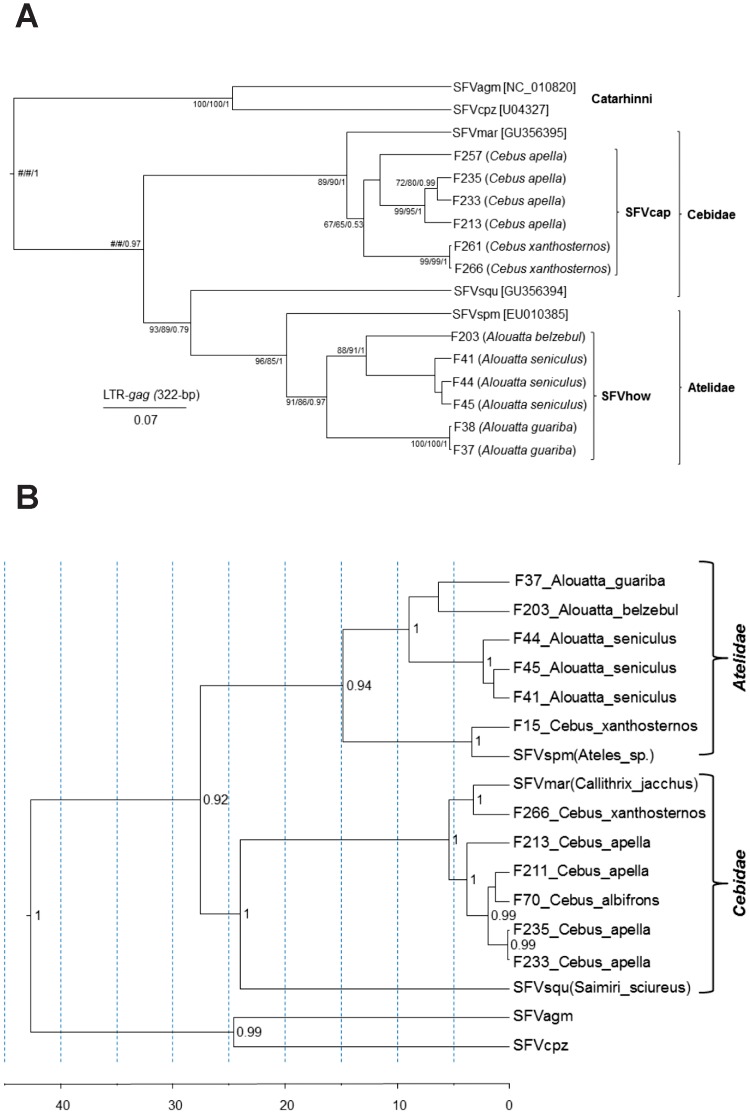
Identification of broad simian foamy virus (SFV) diversity in New World primates (NWPs). Phylogenetic inference of 265-bp SFV long terminal repeat (LTR)/*gag* (*A*) and 276-bp polymerase (*pol)* (*B*) sequences from neotropical primate species. SFV sequences retrieved from GenBank are shown with their respective accession numbers, while the remaining SFV are those generated in the study. The newly characterized SFV lineages infecting capuchins (SFVcap) and howler monkeys (SFVhow) can be seen in both panels. Scale bar for the SFV LTR/*gag* tree is in nucleotide substitutions per site. Statistical support for branch nodes in the LTR/*gag* tree are provided as bootstrap values from neighbor-joining (NJ) and maximum likelihood (ML) methods and posterior probabilities from Bayesian inference (BI) in the order NJ/ML/BI. # indicates statistical support was not provided by the respective program. Topology and divergence dates for the *pol* tree were inferred using a relaxed molecular clock and a Yule tree prior using BEAST v1.6.2. X axis is in millions of years. Posterior probabilities >0.8 are provided at nodes.

Significant substitution saturation was found in the 3^rd^ codon position (cdp) of the SFV *pol* but not the *cytB* alignments, confirming the high levels of diversity detected above. Thus, a shorter 276-bp alignment consisting of the 1^st^ and 2^nd^ cdps from 13 different animals was used for the SFV phylogenies. In addition, phylogenetic relationships of SFV/LTR sequences from 12 different animals was determined. All SFV *pol* and LTR/*gag* sequences were from captive capuchins (*Cebus*) and howler (*Alouatta*) monkeys. Phylogenetic analysis of both regions identified individual clades comprising two novel lineages of SFVs from capuchins (SFVcap) and howler (SFVhow) monkeys in addition to the marmoset (SFVmar), squirrel monkey (SFVsqu), and spider monkey (SFVspm) lineages (Figures 3A and B). The SFV phylogenies strongly resembled those of the *cytB* host sequences with formation of NWM and OWM clades (Figs. 2 and 3). In addition, within the NWM (Platyrrhini) clade the Cebidae and Atelinae SFVs also clustered together as for the *cytB* phylogenies suggestive of SFV host co-evolution (Figs. 2 and 3). Within the howler monkey clades, further structuring of SFV phylogeny by species is also evident with three distinct lineages corresponding to individual howler monkey species (*Alouatta guariba, A. belzebul* and *A. seniculus*) in both the LTR/*gag* and *pol* phylogenies. Similarly, SFVs from two capuchin species (*Cebus apella* and *C. xanthosternos*) formed separate lineages in the LTR/*gag* tree (Fig. 3A), but were mixed with SFVs from *C. albifrons* or spider monkeys in the *pol* tree (Fig. 3B). The LTR/*gag* phylogeny also showed two pairs of viruses from different specimens with no genetic variation in *Cebus xanthosternos* (F261 and F266) and *Alouatta guariba* (F37 and F38) (Fig. 3A). In both cases, these pairs represent animals that shared the same cage and are likely direct transmissions between cagemates. Longer *pol* sequences were not available from animals F261 and F38 for comparison with F266 and F37 to verify the high genetic identity in these pairs in another genomic region, but all four animals were PCR-positive using the generic screening *pol* primers. Similar LTR/*gag* and *pol* tree topologies were inferred using the NJ, ML and BI methods (data not shown).

Interestingly, SFV *pol* sequences from two specimens of *Cebus xanthosternos* (F15 and F266) did not cluster with viruses from the remaining *Cebus* (capuchin) monkeys. Whereas F15 clustered with an SFV from a spider monkey, F266 grouped with SFV from a marmoset (Fig. 3B). Moreover, the placement of F266 outside the SFVcap clade was restricted to the *pol* fragment, whereas this sequence clustered within the capuchin virus group in the LTR/*gag* fragment, indicative of viral recombination, and will require further studies. These two viral strains in animals F15 and F266 likely represent cross-species transmission events, suggesting *Cebus* as a particularly susceptible primate to these viruses. Analysis of host *cytB* sequences from these two animals confirmed they are yellow-breasted capuchins (Fig. 2), eliminating any possibility of their misclassification or other systematic errors.

**Figure 4:**
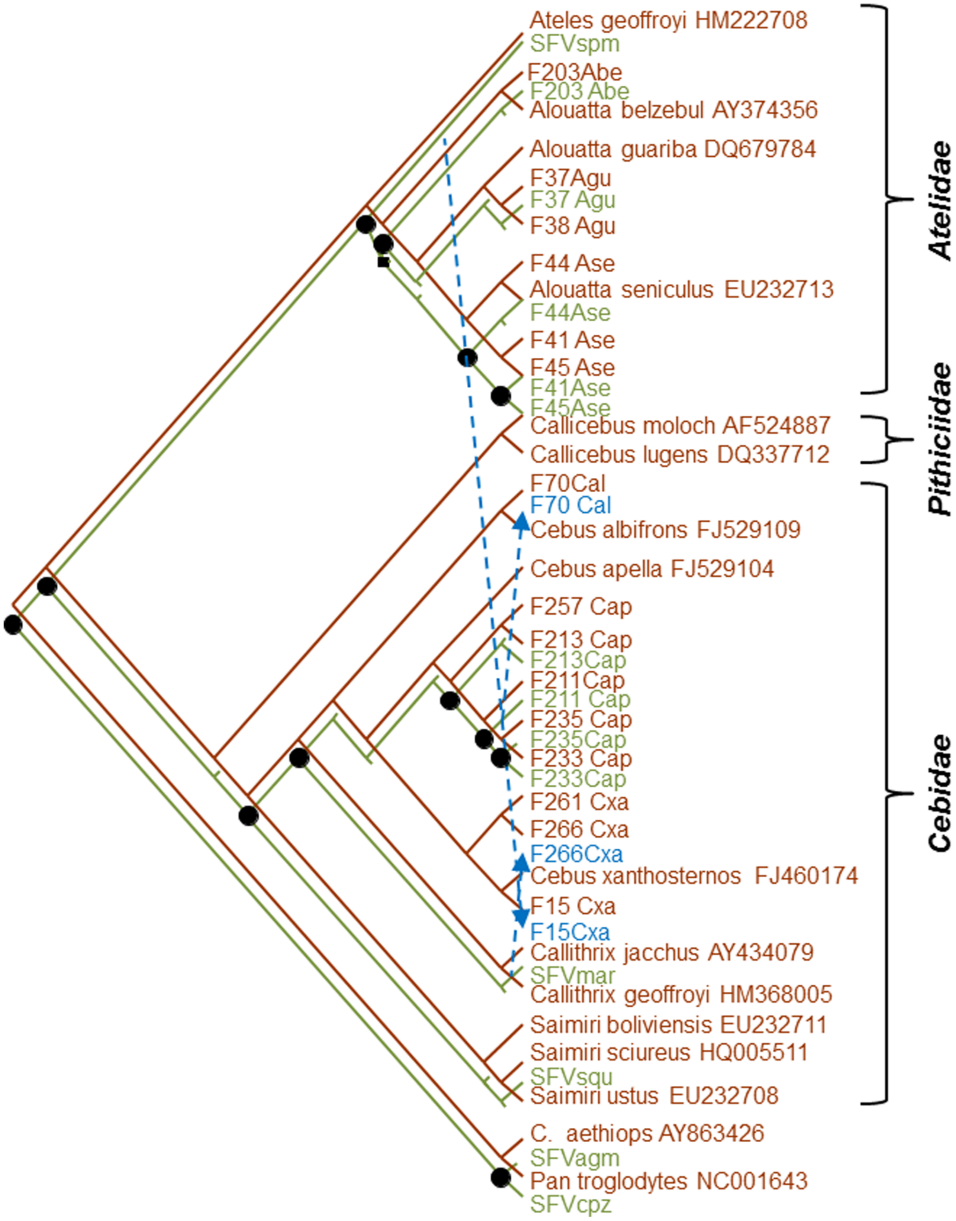
Co-evolutionary relationships of simian foamy virus (SFV) polymerase (*pol*) (green branches and text) and primate cytochrome B (*cytB*) (brown branches and text) Bayesian-inferred phylogenetic trees based on reconciliation analysis. One of nine potentially optimal reconciled trees with 12 cospeciations (black circles), three host switches (blue arrows with dashed lines), 1 duplication (black square), and 15 sorting events (truncated branches without corresponding taxa).

**Figure 5:**
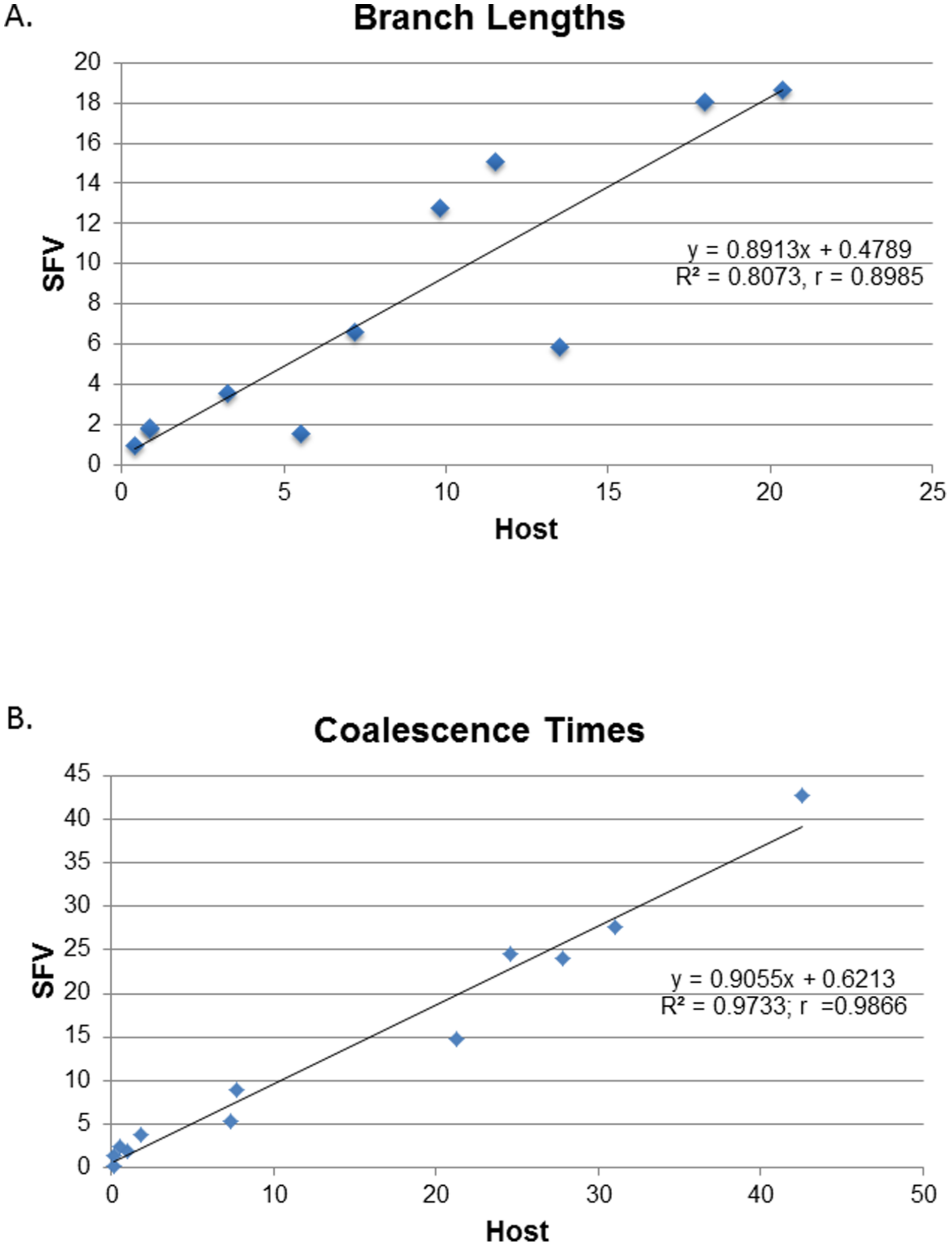
Correlation of (*A*) branch lengths (substitutions per site) and (*B*) coalescence times (genetic distances) of primate cytochrome B and SFV polymerase (*pol*) Bayesian-inferred phylogenetic trees.

To further explore the co-speciation hypothesis and evaluate possible cross-species infection of the two *Cebus xanthosternos*, tree reconciliation analyses were performed and which identified a single and nine optimal reconstructions using the heuristic and exact search options with inference of 11–12 co-speciation events, respectively. Of these, one reconstruction fit the phylogenetic results and specimen histories better with an estimated 12 co-speciation, one duplication, three host switches, and 15 sorting events (Fig. 4) and was strongly supported following randomization of both primate and SFV trees (P<0.00001). The analysis confirmed the SFV from a marmoset and spider monkey switched hosts in two *Cebus xanthosternos* (F15Cxa and F266Cxa) (Fig. 3B). The third host switch was inferred to have occurred from an SFV-infected *C. apella* to a *C. albifrons* (F70) (Fig. 4). We also found strong linear relationships between the branch lengths (r  = 0.8985) and coalescence times (r  = 0.9866) for the host and SFV trees (Fig. 5), indicating that the accumulation of genetic diversity has occurred over an equivalent period in both data sets. Moreover, we found high agreement between the internode divergence times of the SFV and *cytB* trees and the fossil record (Table 4). For example, the TMRCA ranges for the both the SFV and *cytB* Platyhrrini overlap those estimated by others and the fossil record estimate of 20.5–26.5 MYA, which in our analysis is also the Atelidae/Cebidae split since SFV sequences from Pithicidae have not yet been reported [30]. Although fossil record estimates are not available for the NWM families and subfamilies, the inferred divergence date ranges for the SFV and *cytB* are also in general agreement with each other and with those obtained by others (Table 4) [30]. Finally, although the strict molecular clock was strongly rejected (p<0.000001) for both SFV and *cytB* trees, we obtained mean nucleotide substitution rates (nucleotides/site/year) using a relaxed clock (7.79×10^–9^, 95% HPD 4.89×10^–9^–1.16×10^–8^) that were very similar to that previously reported by our group for OWM SFVs (1.7–1.8×10^–8^). The mean substitution rate for the *cytB* sequence was also similar to that of SFV (6.24×10^–9^, 95% HPD 4.0–9.0×10^–9^), which would be expected if both virus and host were cospeciating.

**Table 4:** Time to most recent common ancestor (tMRCA) mean estimates for Haplorrhini and simian foamy virus (SFV) polymerase (*pol)* and simian cytochrome B (*cytB*) sequences^a^.

Branch node	tMRCA SFV *pol*	tMRCA *cytB*	tMRCA simian phylogeny^b^	Fossil estimate^b^
Haplorrhini	42.38 (33.86–51.11)	42.37 (34.26–51.27)	43.47 (38.55–48.36)	43+4.5
Catarrhini	24.33 (15.52–35.17)	24.17 (15.1–35.3)	31.56 (25.66–37.88)	29+6.0
Platyrrhini	28.11(15.02–45.21)	34.58 (20.43–49.7)	24.82 (20.55–29.25)	23.5+3.0
Atelidae	15.55 (6.12–31.21)	20.55 (8.21–37.14)	16.13 (10.52–21.35)	NA^d^
Atelinae	3.4 (0.75–9.27)	ND^c^	11.25 (7.25–15.46)	NA
Alouattinae	9.06 (3.64–18.47)	7.89 (2.4–17.85)	6.03 (3.74–8.57)	NA
Cebinae	3.89 (1.32–8.57)	13.1 (5.96–23.24)	6.00 (3.13–9.35)	NA
Saimirinae	3.37 (0.75–9.27)	5.4 (1.48–12.62)	ND^c^	NA
Callitrichinae	3.21 (0.62–7.8)	2.79 (0.4–8.67)	8.42 (5.72–11.38)	NA

a Using an alignment of 276-bp of 1^st^ and 2^nd^ codon positions for 18 SFV taxa and 500-bp of all codon positions for 31 *cytB* taxa. Million years (MY) ago. Geometric means inferred using Bayesian methods and a relaxed clock; ranges in parentheses are 95% highest posterior density intervals.

b Dating and fossil estimates from Perelman *et al.* 2011 [30].

c ND, not determined.

d NA, not available.

## Discussion

FVs are the only exogenous retroviruses that have been identified in NWPs. However, despite five decades of studying FVs only three SFV variants have been molecularly characterized from platyrrhines [20], [21]. In addition, the diversity of SFV infecting NWP, as well as their dissemination and geographic distribution in nature, is largely unknown. African and Asian primates have been repeatedly shown as sources of zoonotic introduction of SFV into humans [3]–[6], [12], and NWP may also pose a similar risk for such transmission, as these animals are kept as pets around the world and are also hunted and butchered for meat consumption in South America. Herein, we expand significantly our understanding of SFV infection in NWPs. We detected eight distinct SFV lineages in 23 different NWP species comprising all three families of neotropical primates (Atelidae, Cebidae, and Pitheciidae). Six of these eight SFV strains are reported for first time and we molecularly characterized in further detail two novel SFV phylogenetic lineages infecting the *Cebus* (capuchins) and *Alouatta* (howler monkeys) genera.

The average molecular prevalence of SFV in NWP estimated here was substantial yet lower (24%) than those reported in African primates which ranged from 60–83% in wild-living and captive mandrills [6], 86% in wild-living red colobus monkeys [34], to 44–100% in communities of wild chimpanzees [35]. In our study, both captive and wild-caught animals showed consistently similar lower prevalence which may be explained by testing of more juvenile animals in our study, which typically have lower SFV infection rates. Unfortunately, the age at collection was not recorded for the animals analyzed and thus we cannot accurately evaluate the effect of age on our PCR results. The lower prevalence may also be due to low proviral loads or lower sensitivity for detecting highly divergent viruses by screening specimens using our new generic PCR assay. However, this assay was found to be highly sensitive and detected a broad range of SFVs in monkeys within each of the three NWP families. In addition, infection with highly divergent SFVs was confirmed in two families using additional sequence analysis, suggesting that the lower prevalence may be from testing of young animals. Although serum and plasma specimens were not available from animals in this study, supplemental screening of NWPs using serologic assays for detecting SFV infection will enhance the sensitivity to accurately measure the prevalence of SFV in NWP.

Phylogenetic separation of SFV LTR/*gag* and *pol* sequences from six NWP species in two (Atelidae and Cebidae) of the three NWP families mirrored that of their hosts and is consistent with virus-host co-speciation which appears to be a common characteristic of SFV evolution [36], [37]. Detailed reconciliation analyses strongly confirmed the co-evolution of SFVs and NWPs in these two families with significant correlations between host and SFV branch lengths and divergence times. Although we identified SFV in additional genera in each NWP family, the *pol* sequences were too short for phylogenetic resolution within the Platyrrhini to further investigate the co-speciation hypothesis. Molecular dating in our study estimates that the NWP SFV are at least 15 million years old, with a mean of 28 MYA, consistent with fossil records [30] and host sequence divergence date estimates determined in our study. Combined with the finding of an endogenous SFV in the prosimian aye-aye genome [38], our findings are congruent with an ancient evolution of FVs in simians for over 85 million years.

We also identified evidence of cross-species transmission in in two captive *Cebus xanthosternos*, involving different genera or even different families. Cross-species transmission of SFV has also been reported between African primate species [34], [39], but is considered a rare event. Although the different NWP species we analyzed herein are typically kept in separate vivariums, it is possible that these two capuchins were in contact with spider (*Ateles* sp.) and marmoset (*Callithrix* sp.) monkeys during transportation or other circumstances. Many of the animals analyzed herein were from Brazilian Wildlife Department confiscations before they were sent to zoos or primate centers, and therefore co-transportation with other species by wildlife dealers or by the law enforcement teams are not known. *Cebus xanthosternos* and *Ateles* habitats also do not overlap in South America. While the former is restricted to the Caatinga forest of northeastern Brazil, the latter lives in the Amazon region. Both groups inhabit distinct biomes, separated by major rivers and a large distance (compare Figs. 1A and B). Conversely, the observed species-specific structure of the SFVhow in the *Alouatta* genus may reflect the limited habitat overlap of the three *Alouatta* species analyzed, also living in distinct biomes (Fig. 1C), or enhanced species-specific restriction of SFV infection.

Cross-species transmission may also explain the clustering of SFVsqu [GU356394] LTR/*gag* sequences with Atelidae SFV rather than other Cebidae SFV, as in the congruent *pol* and cytB phylogenies. Although the provenance of this squirrel monkey has not been reported in detail and was most likely born in captivity [21], it is possible that it was in contact with Atelidae monkeys or the host taxonomy is incorrect since host sequences are not available for this animal. More parsimonious explanations are long branch attraction due to homoplasy in the sequence and/or poor taxonomic sampling to accurately resolve the SFVsqu phylogeny in the region [40]. The characterization of additional SFV sequences and genomes from other squirrel and spider monkeys will help to clarify the classification of these viruses. We also identified two cases of direct SFV transmission between monkeys living in the same cage, one between two *Cebus xanthosternos* (F261 and F266), and the other between two *Alouatta guariba* (F37 and F38). The SFV LTR/*gag* sequences from each transmission pair were identical. The low intrahost variability of SFV, the smallest seen among complex primate retroviruses [34], is likely a result of limited viral replication in blood [6], [34], [37], which may explain the low divergence observed in these pairs. Recent transmission would also explain such low diversity. The exact route of transmission in these cases is currently unknown but likely occurred from grooming or biting which is known to transmit SFV. Further controlled studies with cohabited, yet discordant specimens would shed additional light on SFV transmission routes in NWP and in primates in general.

None of the NWP studied here presented clinical symptoms typical of retroviral infections at the time of specimen collection, including immunodeficiency, inflammation, neurologic disorders, and malignancies. All screened animals were adults at sample collection, and no age-specific correlations can be drawn from our study. It is known that SFV infection increases with age and especially as animals become adults [35], [39]. While monkey gender does not appear to influence SFV acquisition, duration of SFV infection was not available for animals in our study, and the cross-sectional study design all limit conclusions regarding transmissibility and whether SFV infection in NWP is pathogenic. In natural hosts, SFV infections have been characterized as asymptomatic and persistent. Such nonpathogenic virus-host relationship may be explained by a long co-existence of exogenous SFV in primates. More systematic studies are needed to establish the pathogenesis of SFV infection in NWP and OWP, especially since retroviral infections can take years to present symptoms or may occur in low prevalence in infected animals. For example, AIDS-like disease in mangabeys and chimpanzees naturally infected with SIV can take at least 7 years to manifest and was only diagnosed by close monitoring of the animals [41]–[43].

Despite the absence of a human-specific FV, SFV can easily be transmitted to men and women originating from a broad variety of African and Asian primates [4], [12], [14], [44]–[47]. As there are very few studies investigating the prevalence and geographical distribution of SFV in NWP, the risk of zoonotic transmission of these viruses to humans remains unknown and will warrant future studies. NWP are frequently hunted for food and kept as pets by indigenous populations and locals of the forest regions in South America, and SFV transmission to humans in this scenario is likely similar to that observed in primate hunters in Africa, deserving further investigation. In addition, persons working with NWPs in captivity may be at risk for infection with SFV, including persons in biomedical research centers, zoos, and pet owners in the US and other countries. The high genetic diversity of SFV documented here increases the pool of viral variants that humans are exposed to which may also influence person-to-person transmissibility and disease potential in the new host.

In conclusion, we demonstrate here for the first time a wide range of South American primate species harboring SFV, including the characterization of two novel SFV lineages, SFVcap and SFVhow. Co-speciation between SFV and their hosts in two NWP families was also observed, as well as two cases of cross-species transmission involving primates from distinct genera and families. Finally, we presented here the first estimate on the molecular epidemiology of SFV infection in both captive and wild-living NWP. Further studies are needed to better characterize additional SFV variants, only preliminarily described here, which will improve our knowledge of retroviral infections in platyrrhines. Moreover, the tools and sequences reported here will facilitate an assessment of the risk of zoonotic infection with SFV in persons naturally or occupationally exposed to NWPs.

## Supporting Information

Figure S1 **Inferred phylogenetic relationships of 138-bp polymerase sequences generated by using the diagnostic PCR primers.** The tree was built using Bayesian inference in the program BEAST and a relaxed molecular clock and a Yule tree prior. Posterior probabilities >0.7 are shown at branch nodes. Genera abbreviations are: Ao., *Aotus*; Al., *Alouatta*; C., *Cebus*; Ca., *Callithrix*; Cal., *Callicebus*; Sa. *Saimiri*; Sag., *Saguinis*; Ch., *Chiropotes*; Le., *Leontopithecus*. spm, spider monkey (*Ateles* species), squ, squirrel monkey (*Saimiri* speies), mar, marmoset (*Callothrix jacchus*), agm, African green monkey (*Chlorocebus* species), cpz, chimpanzee (*Pan trogolodytes*).
(TIF)

Table S1 PCR primers for detection of SFV LTR/*gag* matrix and polymerase (*pol*) sequences.
(DOCX)
