# Supplementary figures and images for: Identification and Characterization of Highly Divergent Simian Foamy Viruses in a Wide Range of New World Primates from Brazil
Source: PLoS One. 2013 Jul 3;8(7):e67568. doi: 10.1371/journal.pone.0067568 (PMC3701081; doi:10.1371/journal.pone.0067568)

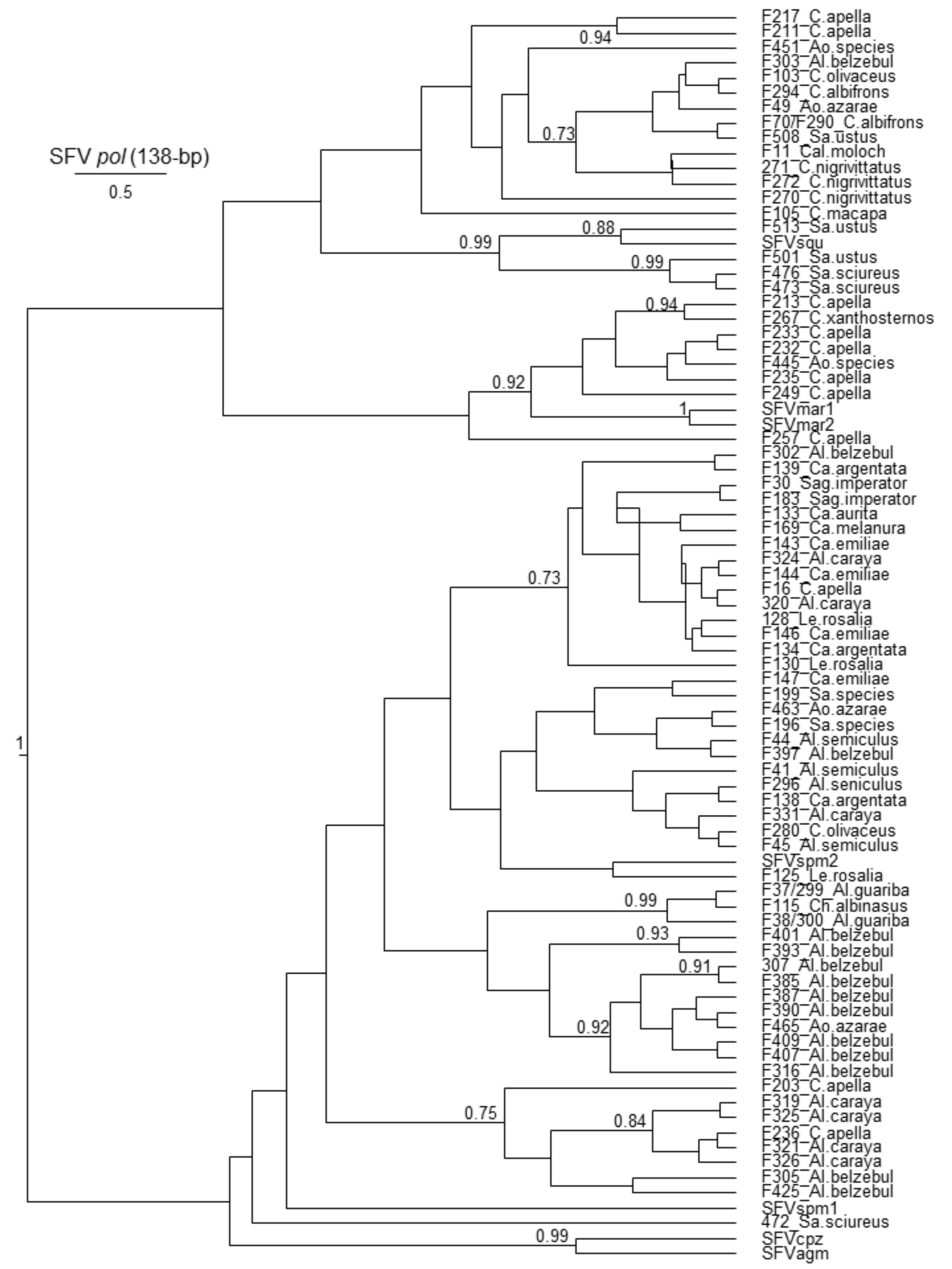

Supplement: Figure S1 — Inferred phylogenetic relationships of 138-bp polymerase sequences generated by using the diagnostic PCR primers. The tree was built using Bayesian inference in the program BEAST and a relaxed molecular clock and a Yule tree prior. Posterior probabilities >0.7 are shown at branch nodes. Genera abbreviations are: Ao., Aotus; Al., Alouatta; C., Cebus; Ca., Callithrix; Cal., Callicebus; Sa. Saimiri; Sag., Saguinis; Ch., Chiropotes; Le., Leontopithecus. spm, spider monkey (Ateles species), squ, squirrel monkey (Saimiri speies), mar, marmoset (Callothrix jacchus), agm, African green monkey (Chlorocebus species), cpz, chimpanzee (Pan trogolodytes). (TIF) [file pone.0067568.s001.tif]
