# Supplementary material for: Identification and Characterization of Highly Divergent Simian Foamy Viruses in a Wide Range of New World Primates from Brazil
Source: PLoS One. 2013 Jul 3;8(7):e67568. doi: 10.1371/journal.pone.0067568 (PMC3701081; doi:10.1371/journal.pone.0067568)
Supplement: Table S1 — PCR primers for detection of SFV LTR/gag matrix and polymerase (pol) sequences. (DOCX) [file pone.0067568.s002.docx]

**Table S1.** PCR primers for detection of SFV LTR/*gag* matrix and polymerase (*pol*) sequences

| Round | Primer | Sequence (5’ 🡪 3’) | Direction | Location^a^ | Size | Annealing Temperature  (35 cycles) |
| --- | --- | --- | --- | --- | --- | --- |
| 1^st^ | SIF5N | tacatggttataccccackaaggctcctcc | Foward | *pol* (5878-5907) | 281-bp | 52^°^C |
|  | SIR5N | aataawggataccactttgtaggtcttcc | Reverse | *pol* (6131-6159) |  |  |
| 2^nd^ | SIP4N | tgcattccgatcaaggatcagcatt | Foward | *pol* (5967-5991) | 192-bp | 55^°^C |
|  | SIR5N | aataawggataccactttgtaggtcttcc | Reverse | *pol* (6131-6159) |  |  |
| 1^st^ | NM 6F | taaaccgcttgyattcgagaacc | Forward | LTR/*gag* (1074-1096) | 607-bp | 37^°^C |
|  | NM 11R | gggcctttrkcwgatggwat | Reverse | LTR/*gag* (1662-1681) |  |  |
| 2^nd^ | NM 7F | tattgttgttgyttctatact | Forward | LTR/*gag* (1106-1126) | 365-bp | 43^°^C |
|  | NM 10R | atgactgaaggatggtgggg | Reverse | LTR/*gag* (1452-1471) |  |  |
| 1^st^ | SFV-1 | gataagttggctryccaaggaagttat | Forward | *po*l (5386-5412) | 601-bp | 46^°^C |
|  | SFV-2 | gctgatccttgatcggartg | Reverse | *pol* (5968-5987) |  |  |
| 2^nd^ | SFV-1 | gataagttggctryccaaggaagttat | Forward | *pol* (5386-5412) | 520-bp | 38^°^C |
|  | SFV-4 | gaaggagctttagtggggta | Reverse | *pol* (5887-5906) |  |  |

^a^Nucleotide positions according to the complete SFV genome from *Ateles* species (GenBank accession # EU010385)*.*
